# Monocolonization of Germ-Free Mice with *Bacteroides fragilis* Protects against Dextran Sulfate Sodium-Induced Acute Colitis

**DOI:** 10.1155/2014/675786

**Published:** 2014-05-29

**Authors:** Chien-Chao Chiu, Yung-Hao Ching, Yu-Chih Wang, Ju-Yun Liu, Yen-Peng Li, Yen-Te Huang, Hsiao-Li Chuang

**Affiliations:** ^1^Graduate Institute of Sports Science, National Taiwan Sport University, Taoyuan 333, Taiwan; ^2^Department of Molecular Biology and Human Genetics, Tzu Chi University, Hualien 970, Taiwan; ^3^Graduate Institute of Medicine, Kaohsiung Medical University, Kaohsiung 807, Taiwan; ^4^National Applied Research Laboratories, National Laboratory Animal Center, Taipei 115, Taiwan; ^5^Department of Microbiology, National Taiwan University College of Medicine, Taipei 115, Taiwan

## Abstract

Ulcerative colitis is inflammatory conditions of the colon caused by interplay of genetic and environmental factors. Previous studies indicated that the gut microflora may be involved in the colonic inflammation. *Bacteroides fragilis* (*BF*) is a Gram-negative anaerobe belonging to the colonic symbiotic. We aimed to investigate the protective role of *BF* in a colitis model induced in germ-free (GF) mice by dextran sulfate sodium (DSS). GF C57BL/6JNarl mice were colonized with *BF* for 28 days before acute colitis was induced by DSS. *BF* colonization significantly increased animal survival by 40%, with less reduction in colon length, and decreased infiltration of inflammatory cells (macrophages and neutrophils) in colon mucosa following challenge with DSS. In addition, *BF* could enhance the mRNA expression of anti-inflammatory-related cytokine such as interleukin 10 (*IL-10*) with polymorphism cytokine *IL-17* and diminish that of proinflammatory-related tumor necrosis factor **α** with inducible nitric oxide synthase in the ulcerated colon. Myeloperoxidase activity was also decreased in *BF*-DSS mice. Taking these together, the *BF* colonization significantly ameliorated DSS-induced colitis by suppressing the activity of inflammatory-related molecules and inducing the production of anti-inflammatory cytokines. *BF* may play an important role in maintaining intestinal immune system homeostasis and regulate inflammatory responses.

## 1. Introduction

Inflammatory bowel diseases including ulcerative colitis and Crohn's disease are disorders of repeated chronic inflammation and mucosal tissue damage of the intestinal tract [1]. Dextran sulfate sodium- (DSS-) induced colonic injury is the most widely used model for studies of the pathogenesis and treatment of ulcerative colitis [2]. Although the etiology of ulcerative colitis remains incompletely understood, the severe and persistent mucosal infiltrations of macrophages and neutrophils in the large intestine are prominent features [3]. In addition, the activity of proinflammatory cytokines such as tumor necrosis factor *α* (TNF-*α*), interleukin 1*β* (IL-1*β*), and IL-6 is increased in the colonic mucosa of patients with ulcerative colitis [4–7]. Previous studies indicated that the IL-17 could promote inflammatory process in* Helicobacter*- and 2,4,6-trinitrobenzene sulfonic acid- (TNBS-) colitis model [8], but several reports also showed IL-17 might have anti-inflammatory function in the DSS- and T-cell-mediated colitis [9, 10].

Clinical and laboratory studies have shown that the density and complexity of the commensal flora provide an important layer of defense against invasion by pathogenic microbes [8, 11, 12]. The gut microbiota can affect colitis development and progression.* Bacteroides *spp. are an important component of the mammalian gut commensal bacteria; the bacteria maintain a complex and generally beneficial relationship with the host by performing immune-regulatory, energy metabolic, and physiologic homeostasis functions. One such species,* Bacteroides fragilis* (*BF*), has profound beneficial effects on the host immune system. Colonized* BF* was sufficient to correct CD4^+^ T-cell defects in spleen and revealed a Th1/Th2 imbalance in germ-free (GF) mice [13]. Moreover,* BF* protected against experimental colitis induced by TNBS or* Helicobacter hepaticus* via interleukin 10 (IL-10) inducing CD4^+^ and Foxp3^+^ regulatory T-cell development [8, 14]. Other genera of* Bacteroides* (*B. vulgatus*,* B. thetaiotaomicron,* and* B. ovatus*) were also found to influence inflammatory bowel disease progression [11, 15, 16].

GF animal models have been a valuable tool in investigating the effects of intestinal bacteria. As reported previously, GF mice showed significant aggravated colonic inflammation as compared with specific pathogen-free mice after DSS treatment [17]. Recent reports also indicated that monocolonization with probiotics (*Escherichia coli* strain Nissle 1917,* Lactobacillus*, and* Bifidobacterium lactis*) or symbiotics (*B. fragilis and B. vulgatus*) could reduce colitis severity in GF mice [16, 18–20]. To date, the protective effect of* BF* on DSS-induced colitis has not been investigated. Therefore, we hypothesized that* BF* colonization in GF mice may protect the colon against the adverse effects of DSS-induced inflammation.

## 2. Materials and Methods

### 2.1. Animals

A total of 60 GF male C57BL/6JNarl mice were used (7-8 weeks old, National Laboratory Animal Center, Taipei, Taiwan). Mice were maintained in a vinyl isolator in a room kept at a constant temperature (21 ± 1°C) and humidity (55–65%) with a 12 h/12 h light/dark schedule. Mice were fed a commercial diet (5010 LabDiet, Purina Mills, St. Louis, MO) and sterile water ad libitum. To confirm GF status, microbiological assays were performed on a monthly basis by culturing feces, bedding, and drinking water in thioglycollate medium (DIFCO, Camarillo, CA). All studies were approved by the Institutional Animal Care and Use Committee (number 2011 M02).

### 2.2. Bacterial Culture and Monocolonization in Mice

*BF* strain NCTC 9343 was obtained from the Food Industry Research and Development Institute (Hsinchu, Taiwan). Bacteria culture was freshly grown overnight in thioglycollate medium (DIFCO, Camarillo, CA) at 37°C on a shaker and then collected by centrifugation (3 min at 2000 g) and washed 3 times with phosphate buffered saline. Pellets were resuspended in 20 mL sterile saline.* BF* was colonized into mice by oral gavage with 5 × 10^7^ colony-formation units (CFUs) of* BF* in 0.5 mL saline. After 28 days of colonization, fecal CFUs were determined by plate count from stool samples and calculated per gram feces. Control mice were treated with the same volume of sterile saline.

### 2.3. Induction of Experimental Colitis in Mice

The acute ulcerative colitis model was induced by the addition of 1% DSS (36–50 kDa, MP Biomedicals, OH, USA) in sterile filtered drinking water in GF mice for 14 days. Animals were randomly divided into the following 4 groups (*n* = 10 each) for treatment: distilled drinking water (GF-WA), 1% DSS in drinking water (GF-DSS),* BF* monocolonization for 28 days and then distilled drinking water (*BF*-WA), or* BF* monocolonization for 28 days and then 1% DSS in drinking water (*BF*-DSS). At day 14 after being treated, mice were killed by CO_2_, and body weight, spleen weight, and colon length were measured. In our pilot study, we found that the treated 1% DSS could not cause any animal death in 2-week experimental period. In order to evaluate survival rates of GF-mice colonized with BF that improve 2% DSS caused animal death. Additional 10 GF mice and 10* BF* mice in each group were treated with 2% DSS for 14 days.

### 2.4. Peripheral Blood Cell Analysis

Blood was collected by intracardiac puncture and treated with ethylenediaminetetraacetate (EDTA). Total blood cells, differential leukocytes, erythrocytes, hemoglobin level, platelets, lymphocytes, and neutrophils were measured by use of the Bayer Hematology System (Bayer, ADVIA 2010).

### 2.5. Occult Blood Assessment in Stools

The presence of blood in stools was assessed by the occult blood reagent method (Shih-Yung Medical, Taipei) and scored on a 0- to 4-point scale: 0: negative; 1: faintly blue; 2: moderately blue; 3: dark blue; and 4: fecal blood visible to the eye.

### 2.6. Histopathology

Mouse colons were fixed in 10% neutral buffered formalin, embedded in paraffin by a standard protocol, then cut into 4 *μ*m sections, stained with hematoxylin and eosin (H&E), and assessed by light microscopy. Colon sections were scored as grade 0: normal mucosa; grade 1: infiltration of inflammatory cells; grade 2: shortening of the crypt; grade 3: crypt loss; and grade 4: destruction of epithelial cells (ulceration and erosion) by the same veterinary pathologist blinded reading.

### 2.7. Immunohistochemical Analysis

Paraffin colon sections were dewaxed, rehydrated and underwent antigen retrieval, and then incubated with 3% H_2_O_2_ to eliminate endogenous peroxidase activity. Sections were incubated with 10% skin milk to reduce nonspecific reactions and incubated overnight with the rat anti-mouse monoclonal antibodies F4/80 (1 : 50; BioLegend, San Diego, CA) or goat anti-mouse polyclonal Ly-6G^+^ (1 : 100; Santa Cruz Biotechnology, Santa Cruz, USA) and then with horseradish peroxidase-conjugated anti-rat or anti-goat antibodies (HRP Polymer Conjugate, Invitrogen, CA); then signals were detected by adding chromogenic substrate (AEC). Sections were rinsed with deionized water, counterstained with hematoxylin, and mounted for histological analysis. The numbers of F4/80- and Ly-6G^+^-positive cells at colon tissue in the GF-DSS and* BF*-DSS group (*n* = 3 per each group) were quantitated by 200x magnification.

### 2.8. Real-Time PCR Analysis of Inflammatory-Related Genes

Total RNA was isolated from colon tissue by use of the RNeasy Minikit (Qiagen, Hilden, Germany). First-strand complementary DNA was synthesized by use of the Transcriptor First Strand cDNA synthesis kit (Roche Diagnostics GmbH). Quantitative real-time PCR reactions involved the TaqMan gene expression assay (Universal Probe Library, Roche Diagnostics GmbH) with LightCycler 1.5 (Roche Diagnostics GmbH) as follows: 95°C for 10 min, followed by 40 cycles of 95°C for 10 sec, 60°C for 25 sec, and 40°C for 30 sec. *β*-Actin was an internal control and nuclease-free water a negative control. The sequences of primers used for analysis are in Table 1. The comparative Ct method was used to evaluate relative mRNA levels in colon tissue.

### 2.9. Myeloperoxidase Activity Assay

Neutrophil accumulation in the colon tissue was measured by myeloperoxidase (MPO) activity. Samples were suspended in 50 mM phosphate buffer containing 0.5% hexadecyltrimethylammonium bromide (pH = 6.0) at a tissue concentration of 50 mg/mL. Samples were homogenized by use of a Polytron homogenizer and centrifuged at 11,000 g for 15 min at 4°C. MPO activity in the supernatant was determined by adding 100 *μ*L of the supernatant to 2.9 mL of 50 mM phosphate buffer (pH 6.0) containing 0.167 mg/mL O-dianisidine hydrochloride and 0.0005% (wt/vol) H_2_O_2_. The change in absorbance at 460 nm over 3 min was measured. One unit of MPO activity was defined as that which would convert 1 *μ*mol of H_2_O_2_ to water in 1 min at 25°C. This part of the procedure was slightly modified from a previous report [19].

### 2.10. Statistical Analysis

Results are presented as mean ± SD. Differences between groups were examined by Student's *t*-test. *P* < 0.05 was considered statistically significant. The Kaplan-Meier survival analysis with log-rank test was applied for comparison of survival times between the different groups.

## 3. Results

### 3.1. *BF* Enhanced Mouse Survival with 2% DSS Challenge

**Figure 1:**
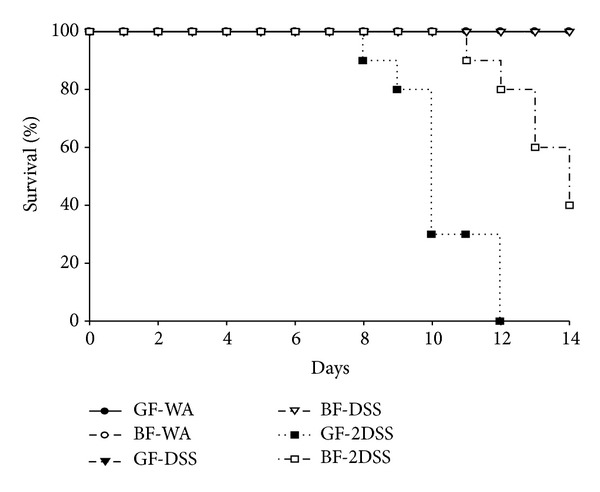
Kaplan-Meier survival curves of germ-free (GF) mice treated with 1% and 2% dextran sulfate sodium (DSS) and* Bacteroides fragilis* (*BF*) (*n* = 10). WA: water.

After colonization of GF mice with* BF* for 28 days, 1 × 10^11^ CFU/g bacteria were detected in feces component of* BF* group animals. Clinical observations confirmed that this bacterium did not cause any colitis signs such as rectal bleeding or diarrhea. Treatment with 2% DSS caused 100% mortality in GF mice from day 8 to day 12. However, the survival rate of the* BF* colonized-mice was significantly higher than GF mice challenge with the same dose of DSS (Figure 1).

### 3.2. *BF* Improved the DSS-Induced Disease Symptoms

As shown in Table 2, a significant decrease in body weight was observed in the GF-DSS group as compared with the GF-WA group by 1% DSS administration for 14 days. The body weight has no significant differences between the* BF*-WA and the* BF*-DSS mice. Spleen weight and ratio of spleen to body weight were significantly higher in GF-DSS than in* BF*-DSS mice. The severity of colitis caused by 1% DSS was associated with significantly shorter colon length in GF-DSS than in GF-WA mice (*P* < 0.05). In contrast,* BF*-DSS colons length was longer than GF-DSS colons but still shorter than GF-WA and* BF*-WA groups. Fecal occult blood scores were significantly increased in GF-DSS mice as compared with controls (*P* < 0.05) but reduced in* BF*-DSS mice (Table 2).

### 3.3. Complete Blood Count Studies

At 14 days after administration of 1% DSS in GF mice, animals became anemic, as indicated by decreased erythrocyte count, haemoglobin level, and haematocrit value (*P* < 0.05). Interestingly, the erythrocyte count, haemoglobin level, and haematocrit value were higher for* BF*-DSS than GF-DSS mice. On the other hand, leukocyte counts with lymphocytes and monocytes were significantly lower for* BF*-DSS than GF-DSS mice. There was not any difference in the leukocyte counts between* BF*-WA and GF-WA mice (see Table 3).

### 3.4. *BF* Ameliorated the Severity of DSS-Induced Histopathologic Changes

**Figure 2:**
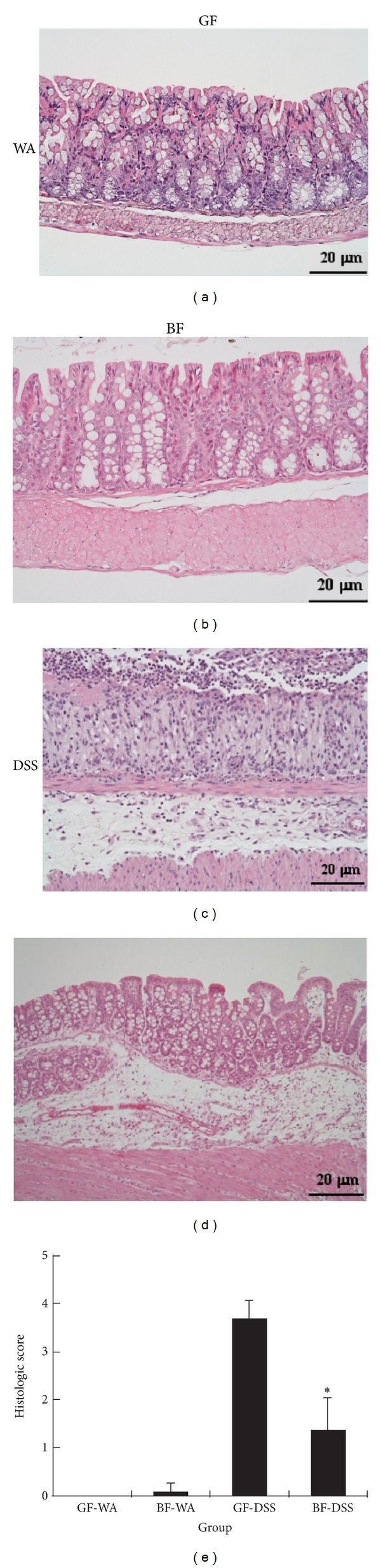
Histopathology of the effect of* BF* monocolonization on DSS-induced colon damage in mice at day 14 after DSS administration. (a) GF-water (GF-WA), (b)* Bacteroides fragilis*-water (*BF*-WA), (c) GF-DSS, (d)* BF*-DSS, and (e) quantification. *P < 0.05 compared with GF-DSS. H&E, magnification ×200, Bar = 20 *μ*m.

On histopathology, GF-DSS colons showed severe mucosa epithelial ulceration (typically affecting more than 75% of the colonial epithelium surface), basal crypt loss, and goblet cell depletion predominantly in the distal colon and rectum (Figure 2). Inflammatory cells, mainly macrophages and neutrophils, appeared to infiltrate into the lamina propria and submucosa. In addition, edema was present between the mucosa and muscular layers of the colon. The mean colon damage score for GF-DSS mice was 3.67 ± 0.41 on a scale of 0 to 4. In contrast,* BF*-DSS colons tissue showed slight/moderate inflammatory infiltration of mononuclear cells in the lamina propria with mucosal architecture mostly intact and slight edema in the submucosa. The mean colon damage score was 1.33 ± 0.68, lower than for GF-DSS colons (*P* < 0.01). Thus,* BF* may protect against the development and progression of DSS-induced colitis in GF mice.

### 3.5. Inflammatory Cells were Decreased in DSS-Colitis by Monocolonization with* BF*

**Figure 3:**
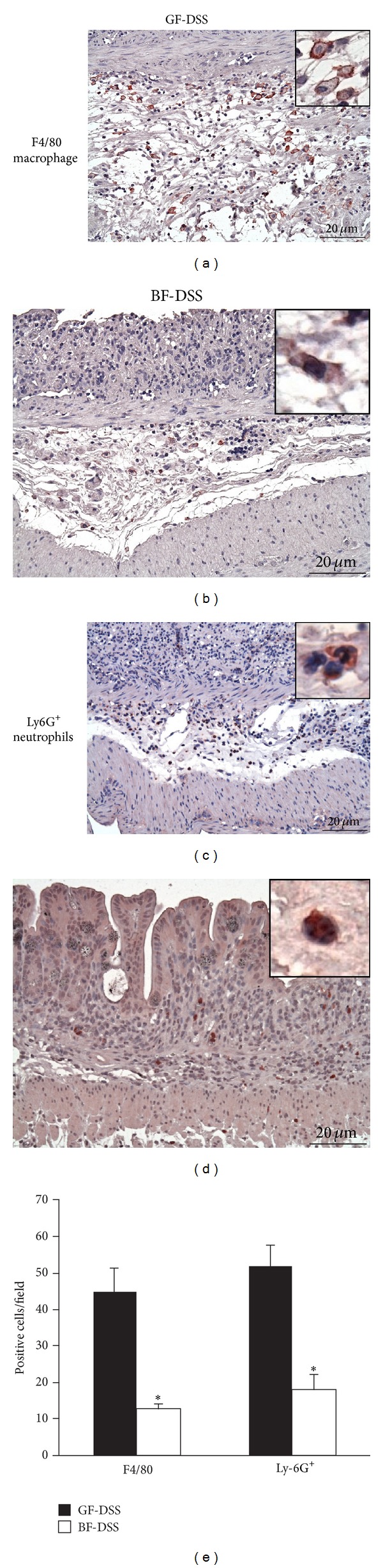
Immunostaining for F4/80 (macrophages) and Ly6G^+^ (neutrophils) in colon tissue sections. (a, c) GF-DSS and (b, d)* BF*-DSS. (e) The numbers of F4/80- and Ly-6G^+^-positive cells were counted in the GF-DSS and* BF*-DSS group (*n* = 3 per each group). *P < 0.05 compared with GF-DSS. H&E, magnification ×200, Bar = 20 *μ*m.

The cellular composition of the inflamed colon was analyzed by immunohistochemistry staining. The number of macrophages (F4/80) and granulocytes (Ly-6G^+^) in the lamina propria and submucosal was assessed. Fourteen days after DSS exposure to the GF mice, a large number of F4/80-positive macrophages and Ly6G^+^-positive neutrophils were found in the lamina propria indicating serious inflammatory response (Figure 3). In contrast to the GF-DSS group, F4/80- (GF-DSS: 45 ± 6 versus* BF*-DSS: 12 ± 1) and Ly6G^+^- (GF-DSS: 52 ± 5 versus* BF*-DSS: 18 ± 4) positive cells decreased in the colon tissue of* BF*-DSS group. A few of F4/80- or Ly6G^+^-positive cells were observed in GF-WA and* BF*-WA groups.

### 3.6. Inflammatory- or Anti-Inflammatory-Related Gene Expression in Colonic Tissue

**Figure 4:**
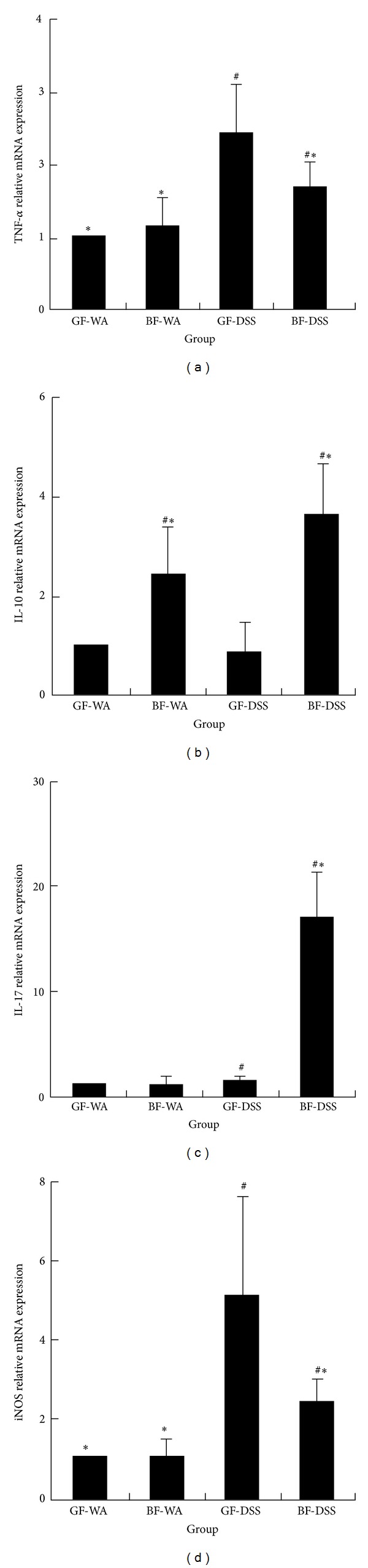
The expression of inflammatory-related genes with DSS-induced colitis. (a) TNF-*α*, (b) IL-10, (c) IL-17, and (d) iNOS. ^#^ *P* < 0.05 compared with GF-WA; *P < 0.05 compared with GF-DSS.

To evaluate expression pattern of cytokines and proinflammatory molecules during the process of DSS colitis in the colons between the experimental and control groups, real-time RT-PCR was performed. As shown in Figure 4, the significantly reduced the gene expression of* TNF-*α** and* iNOS* in the* BF*-DSS mice compared with GF-DSS mice. In contrast, the expression of* IL-10* and* IL-17* was increased more in the* BF*-DSS group than in the GF-DSS group. There were no significant differences between the GF-WA and* BF*-WA groups.

### 3.7. MPO Activity Was Attenuated by* BF* Colonization

**Figure 5:**
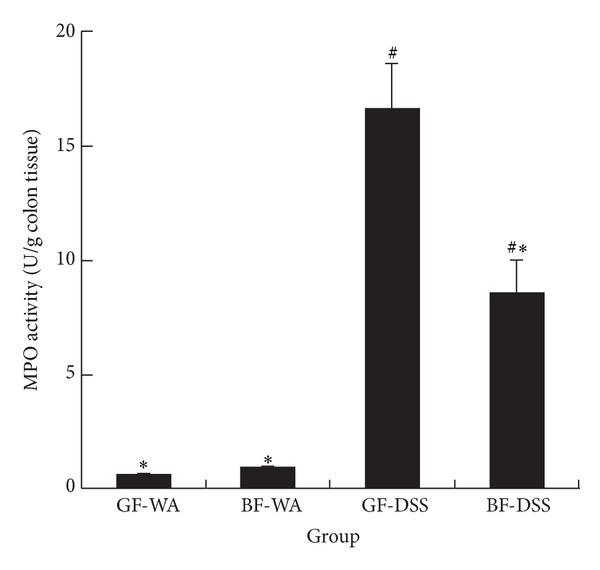
MPO activity in the colon. ^#^ *P* < 0.05 compared with GF-WA; *P < 0.05 compared with GF-DSS.

MPO activity is a marker of neutrophil content and is upregulated in inflammatory tissue. With DSS, the increased MPO activity was nevertheless lower in* BF*-DSS than in GF-DSS colons (8.5 ± 1.54 versus 16.5 ± 2.12 U/g colon tissue). These effects were not observed in GF-WA and* BF*-WA colons (Figure 5).

## 4. Discussion

Ulcerative colitis is a chronic nonspecific inflammatory disorder with unknown etiology. Some intestinal microbes may promote or reduce intestinal inflammation [16, 21, 22]. In this study, we used GF mice and gnotobiotic technology to evaluate the inflammation-protective effect of* BF* on ulcerative colitis development. Administration of 1% DSS to GF mice induced severe colitis in the distal colon. In contrast, mildly basal crypt loss, slightly inflammatory cell infiltration and lamina propria edema in* BF*-DSS mice. In addition,* BF* monocolonization could suppress the expression of inflammatory molecules and induce that of anti-inflammatory cytokines.

We used 2% DSS-induced 100% death in GF mice at 8 to 12 days after exposure, which agrees with previous reports of different inbred strains of mice in the GF condition [17, 23]. These findings were similar to those of Bylund-Fellenius et al., who found higher mortality in GF mice than conventional mice with the same dose of DSS-induced severe colitis [24]. Interestingly, the survival time was prolonged and survival rate increased for* BF*-DSS mice, which suggests the protective effect of* BF*.

We then investigated the protective effect of* BF* on clinical signs during DSS-induced colitis. GF-DSS mice showed prominent weight loss (~5%) due to colonic inflammation as compared with GF-WA mice. Body weight reduction with DSS administration was ameliorated in* BF* versus GF mice (2.8% versus 4.9%), fecal occult blood findings were decreased, colon length was increased, and splenomegaly reduced. Therefore, the* BF* could affect the clinical symptoms of DSS-induced colitis. Our data is consistent with previous study showing that monocolonizationwith* BF *could rescue the immune hyperactivation and induce anti-inflammatory properties to alleviate colitis in GF mice [8].

In patients with inflammatory bowel disease, the innate immune response plays a critical role in disease progression. Activated macrophages and neutrophils secrete proinflammatory cytokines such as TNF-*α*, IL-1*β*, and IL-6 to regulate the inflammatory response in the colonic mucosa of patients with ulcerative colitis [4–6]. Here, we report that* BF* treatment decreased the production of the proinflammatory cytokine TNF-*α* and increased that of the anti-inflammatory cytokine IL-10 in the colon.* BF* could influence both the induction and effector functions of the mucosal immune system. We found significantly reduced TNF-*α* production locally, despite the clear differences in colitis severity between* BF*-DSS and GF-DSS mice. This finding is similar to the administration of probiotics downregulating TNF-*α* production [18, 19, 25].

IL-17 is a pleiotropic cytokine that plays pivotal role in the pro- and anti-inflammatory responses in various tissues and different colitis models [7–10]. Previous studies demonstrated that the development of DSS-induced colitis was enhanced by the administration of an anti-IL-17 neutralizing monoclonal antibody, which suggests that IL-17 plays an anti-inflammatory role in mediating the initiation and progression of inflammation in DSS colitis [9]. The specificity was also confirmed by the finding that the effects of an anti-IL-17 monoclonal antibody were abolished by the administration of recombinant mouse IL-17. In present study, monocolonization of BF without DSS treatment could not induce IL-17 expression in the colon tissue. Interestingly, the IL-17 mRNA expression in the colon tissue was significantly higher in the* BF*-DSS than GF-DSS group. According to above results and references, we presumed that elevation of IL-17 gene expression might involve the* BF* bacteria improved DSS-colonic inflammation. However, further studies are required to evaluate the role of IL-17 in* BF* ameliorated DSS colitis on GF condition. IL-10 is a pluripotent cytokine and the most important anti-inflammatory cytokine found within the higher vertebrate immune response. The crucial role of IL-10 in the prevention of inflammatory bowel disease has been demonstrated by experiments in IL-10-deficient mice and daily systemic administration of recombinant IL-10 [26]. Here, we report that* BF* colonization could increase the production of IL-10 in the large intestine. This increase can be explained by the compensatory mechanism in response to markedly increased production of proinflammatory cytokines, primarily TNF-*α*, whereby an increase in IL-10 level is stimulated by TNF-*α* and may in turn inhibit production of TNF-*α* to maintain the balance of pro- and anti-inflammatory cytokines.

An increased production of iNOS has been found in models of inflammatory bowel disease [27]. Impaired NO production in intestine might have a beneficial effect on the progression of such disease. Many studies involving iNOS inhibitors or iNOS-deficient mice have shown amelioration of DSS-induced colitis, which suggests a possible involvement of an inflammatory molecule (iNOS) in the progression of DSS colitis [28, 29]. We found that administration of* BF* also significantly reduced the gene expression of iNOS.

MPO is an indicator of neutrophil infiltration. MPO activity was higher in our GF-DSS than* BF*-DSS mice, which agrees with the severe intestinal inflammation in the former mice [30]. In contrast, the enzyme activity was less increased in* BF*-DSS than GF-DSS colons, which confirms only mild infiltration of neutrophils into colonic tissue.

In conclusion, our data show that DSS induces severity of innate proinflammatory immune response in GF mouse colons that is downregulated by monocolonization with* BF*. This is the first evidence of the protective effects of* BF* in GF-DSS animal model and might provide a novel therapeutic approach for inflammatory bowel disease.

## Figures and Tables

**Table 1:** Real-time PCR primers used in this study.

Gene (NCBI ID)	Orientation	Sequence (5′-3′)	UPL
TNF-*α*	Forward	tgcctatgtctcagcctcttc	49
(NM_013693.2)	Reverse	gaggccatttgggaacttct	
IL-10	Forward	cagagccacatgctcctaga	41
(NM_010548.1)	Reverse	tgtccagctggtcctttgtt	
IL-17	Forward	cagggagagcttcatctgtgt	74
(NM_010552.3)	Reverse	gctgagctttgagggatgat	
iNOS	Forward	gggctgtcacggagatca	76
(NM_010927.3)	Reverse	ccatgatggtcacattctgc	
*β*-Actin	Forward	ctaaggccaaccgtgaaaag	64
(NM_007393.3)	Reverse	accagaggcatacagggaca	

**Table 2:** Effect of DSS on body weight, spleen weight, ratio of spleen to body weight, and colon length in each group.

	GF-WA	BF-WA	GF-DSS	BF-DSS
Body weight (g)	28.07 ± 0.82	25.87 ± 0.89	26.69 ± 0.73^#^	25.12 ± 0.73
Spleen weight (g)	0.064 ± 0.009	0.062 ± 0.012	0.098 ± 0.023^#^	0.070 ± 0.009*
Spleen/body weight (%)	0.22 ± 0.03	0.23 ± 0.06	0.37 ± 0.06^#^	0.28 ± 0.09*
Colon length (cm)	8.4 ± 0.3	7.6 ± 0.4	6.3 ± 0.4^#^	6.8 ± 0.4*
Occult index	0 ± 0	0 ± 0	3.8 ± 0.5^#^	1.3 ± 0.5*

WA: water.

^#^ *P* < 0.05 compared with GF-WA; *P < 0.05 compared with GF-DSS.

**Table 3:** Effect of DSS on haematological characteristics of GF and *BF* mice.

	GF-WA	BF-WA	GF-DSS	BF-DSS

Leukocytes (10^3^ cells/*μ*L)	7.0 ± 1.04	7.1 ± 1.02	10.8 ± 1.73^#^	6.8 ± 0.61*
Neutrophils	0.7 ± 0.11	0.7 ± 0.26	1.0 ± 0.14^#^	0.8 ± 0.17
Lymphocyte	5.5 ± 1.31	4.3 ± 1.2	8.9 ± 1.6^#^	4.8 ± 0.17*
Monocyte	0.08 ± 0.03	0.05 ± 0.02	0.19 ± 0.05^#^	0.06 ± 0.01*
Erythrocytes (10^6^ cells/*μ*L)	10.1 ± 0.44	10.2 ± 0.46	6.2 ± 1.40^#^	8.9 ± 0.83*
Haemoglobin level (g/dL)	14.4 ± 0.34	14.3 ± 0.73	8.4 ± 1.89^#^	12.9 ± 1.24*
Haematocrit value (%)	48.8 ± 2.13	49.0 ± 2.50	30.3 ± 4.80^#^	41.2 ± 3.86*
Platelet count (/*μ*L)	1394.6 ± 170.93	1632.6 ± 153.32	1393.7 ± 315.47	1554.9 ± 302.49

^#^ *P* < 0.05 compared with GF-WA; *P < 0.05 compared with GF-DSS.
